# Supplementary material for: Delicate Ferromagnetism in MnBi6Te10
Source: Nano Lett. 2022 Oct 31;22(24):9815–22. doi: 10.1021/acs.nanolett.2c02500 (PMC9801432; doi:10.1021/acs.nanolett.2c02500)
Supplement: Supplementary file 1 — nl2c02500_si_001.pdf [file nl2c02500_si_001.pdf]

Supporting Information (SI)

Delicate Ferromagnetism in $MnBi_6Te_{10}$

Chenhui Yan[1], Yanglin Zhu[2], Leixin Miao[3], Sebastian Fernandez-Mulligan[1], Emanuel Green[1], Ruobing Mei[2], Hengxin Tan[4], Binghai Yan[4], Chao-Xing Liu[2], Nasim Alem[3], Zhiqiang Mao[2] & Shuolong Yang[1]*

[1]Pritzker School of Molecular Engineering, University of Chicago, Chicago, Illinois 60637, USA

[2]Department of Physics, Pennsylvania State University, University Park, State College, Pennsylvania 16802, USA

[3]Department of Materials Science and Engineering, The Pennsylvania State University, University Park, PA, 16802, USA

[4]Department of Condensed Matter Physics, Weizmann Institute of Science, Rehovot 7610001, Israel

*yangsl@uchicago.edu

SI Note 1: Defects and doping

It is a general trend that the ARPES spectrum becomes less electron-doped with an increasing thickness of BT in $MnBi_{2n}Te_{3n+1}$.[1,2] Meanwhile, the doping change from the 1-BT termination to the 2-BT termination is much more dramatic in FM $MnBi_6Te_{10}$. While a clear mechanism is yet to be elucidated, we speculate that it is rooted in the particular defect configuration in FM $MnBi_6Te_{10}$. There are at least three kinds of defects that can lead to hole doping in Bi_2Te_3-derived materials: Bi-Te antisites,[4] Mn^{2+}-Bi^{3+} antisites, and Bi vacancies. Microscopically, the higher Mn vacancies in FM $MnBi_6Te_{10}$ would allow more Bi atoms to migrate from the BT layers to the MBT layer during the crystal growth. This would lead to a higher concentration of Mn^{2+}-Bi^{3+} antisites and Bi vacancies on the BT side of the MBT/BT interface. The 2-BT termination is formed by the cleavage at the MBT/BT interface, and is hence more hole-doped as compared to the counterpart in AFM $MnBi_6Te_{10}$. The 1-BT termination is less influenced, as the exposed surface is further away from the MBT/BT interface. Our STEM-EDX measurements (SI Table 1) confirm both the higher Mn vacancies and higher Bi vacancies in FM $MnBi_6Te_{10}$, which provides indirect support of this Bi-migration induced hole doping. The overall concentration of Mn^{2+}-Bi^{3+} antisites is lower in FM $MnBi_6Te_{10}$, but we suspect that the Bi vacancies on the 2-BT termination of FM $MnBi_6Te_{10}$ may play a more dominant role due to the more drastic change of cation valence.

To fully understand the defect-induced doping effect, one needs to perform atomically-resolved scanning tunneling microscopy, which is beyond the current scope of our work.

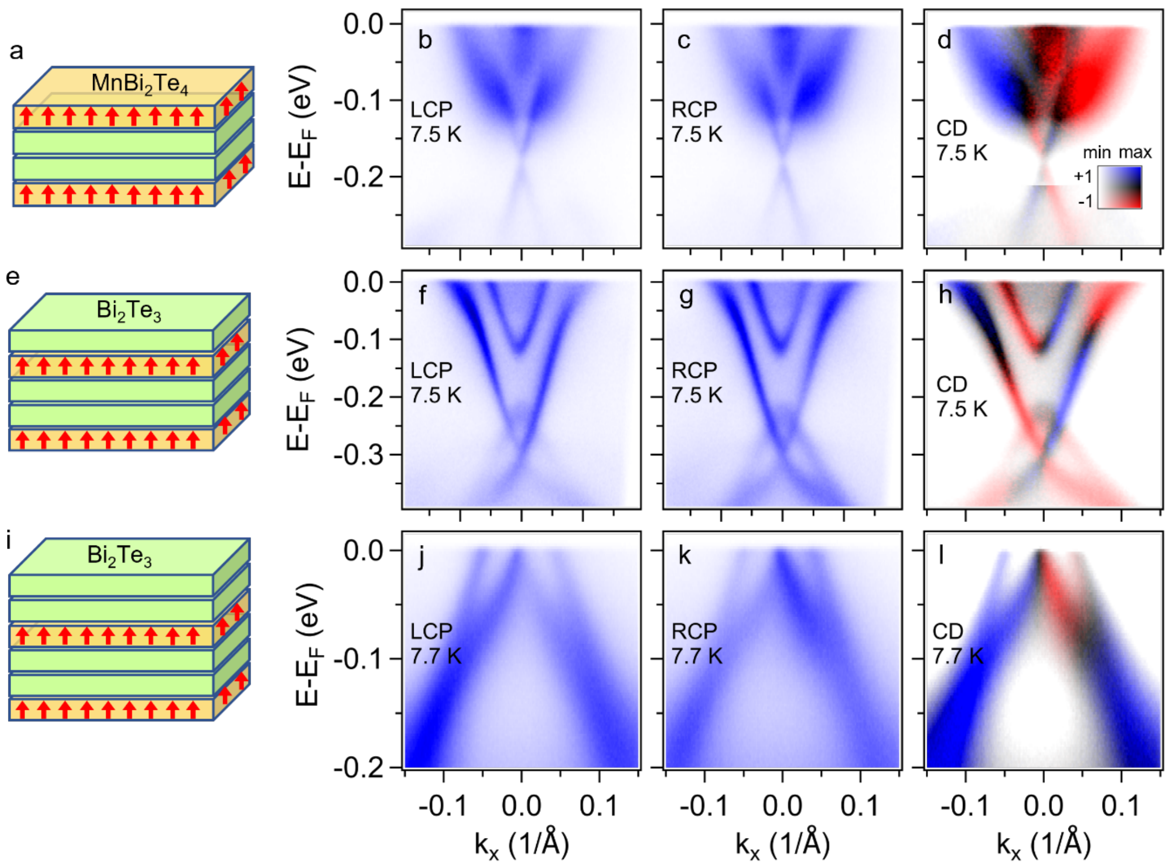

SI Figure 1. Circular dichroism in the ARPES spectra of ferromagnetic $MnBi_6Te_{10}$. (a) Schematic drawing of the $MnBi_2Te_4$ (MBT) termination. (b, c) Energy-momentum spectra along $\bar{\Gamma} - \bar{M}$ measured with (b) the left circular polarization (LCP) and (c) the right circular polarization (RCP). (d) Circular dichroism map. We adopt a 2D color scale where the red-blue contrast represents the LCP-RCP spectral difference normalized by their sum, and the overall saturation represents the spectral intensity. The intensity scale is adjusted for the lower branch of the Dirac cone to account for the much weaker spectral intensity. The counterpart results for the 1-Bi_2Te_3 (1-BT) termination are plotted in (e-h). The counterpart results for the 2-Bi_2Te_3 (2-BT) termination are plotted in (i-l).

SI Note 2: A Side-by-side comparison of FM and AFM $MnBi_6Te_{10}$ using the temperature evolutions of the TSS's on the MBT termination.

To quantify the magnetically induced gap in FM $MnBi_6Te_{10}$, we extract the TSS dispersion by fitting momentum distribution curves (MDCs) using Lorentzian peaks (red dots in SI Figure 2a), and subsequently fit the band dispersion to a standard model incorporating potential band gaps and band curvatures.[5] The gap size is extracted as a function of temperature (SI Figure 2c), which is in turn fitted to a power law function[2]: $\Delta = (1 - T/T_0)^{2\beta}$, resulting in $T_0 = 12.7 \pm 1.4$ K and $\beta = 0.11 \pm 0.09$. The coincidence between the gap closing temperature T_0 and the magnetic T_c directly demonstrates that the gap opening originates from ferromagnetism. Meanwhile, inspection of the 2D curvature plots (SI Figure 2b) suggests that a finite gap above T_c may not be excluded, potentially due to extrinsic reasons such as local impurities. For AFM $MnBi_6Te_{10}$, a gapless TSS persists to above the AFM transition temperature, as demonstrated by both the energy-momentum spectra (SI Figure 2d, e) and the EDCs (SI Figure 2f).

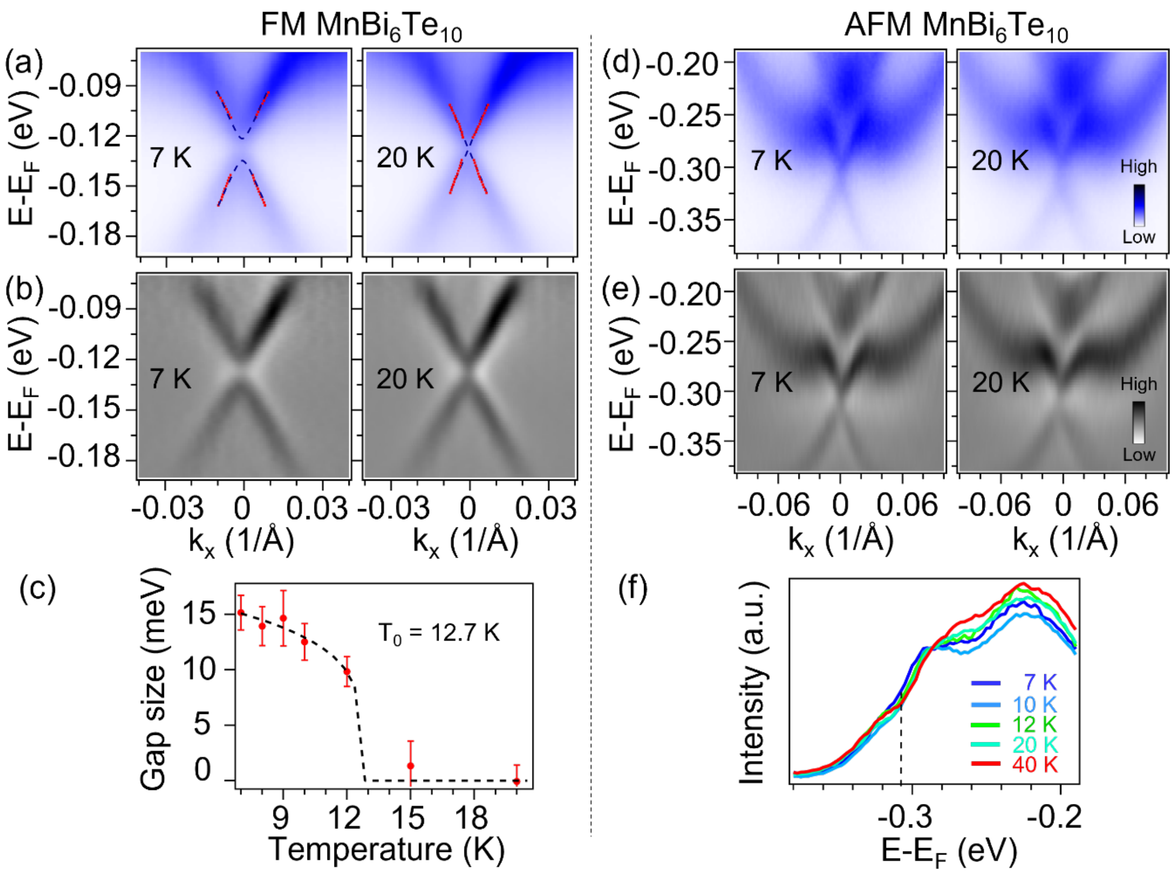

SI Figure 2. Temperature evolution of topological surface states on the MBT termination of $MnBi_6Te_{10}$. (a) Zoomed-in ARPES spectra for FM $MnBi_6Te_{10}$ taken at 7 K (left) and 20 K (right). The overlaid red dots are extracted by fitting momentum distribution curves. The blue dashed lines represent extracted band dispersions.[5] (b) 2D curvature maps of the spectra in (a).[6] (c) Extracted gap magnitude as a function of temperature, which is fitted to $\Delta = (1 - T/T_0)^{2\beta}$. (d) Zoomed-in ARPES spectra for AFM $MnBi_6Te_{10}$ taken at 7 K (left) and 20 K (right). (e) 2D curvature maps of the spectra in (d). (f) Temperature evolution of energy distribution curves at $\bar{\Gamma}$. The black dashed line marks the Dirac point energy.

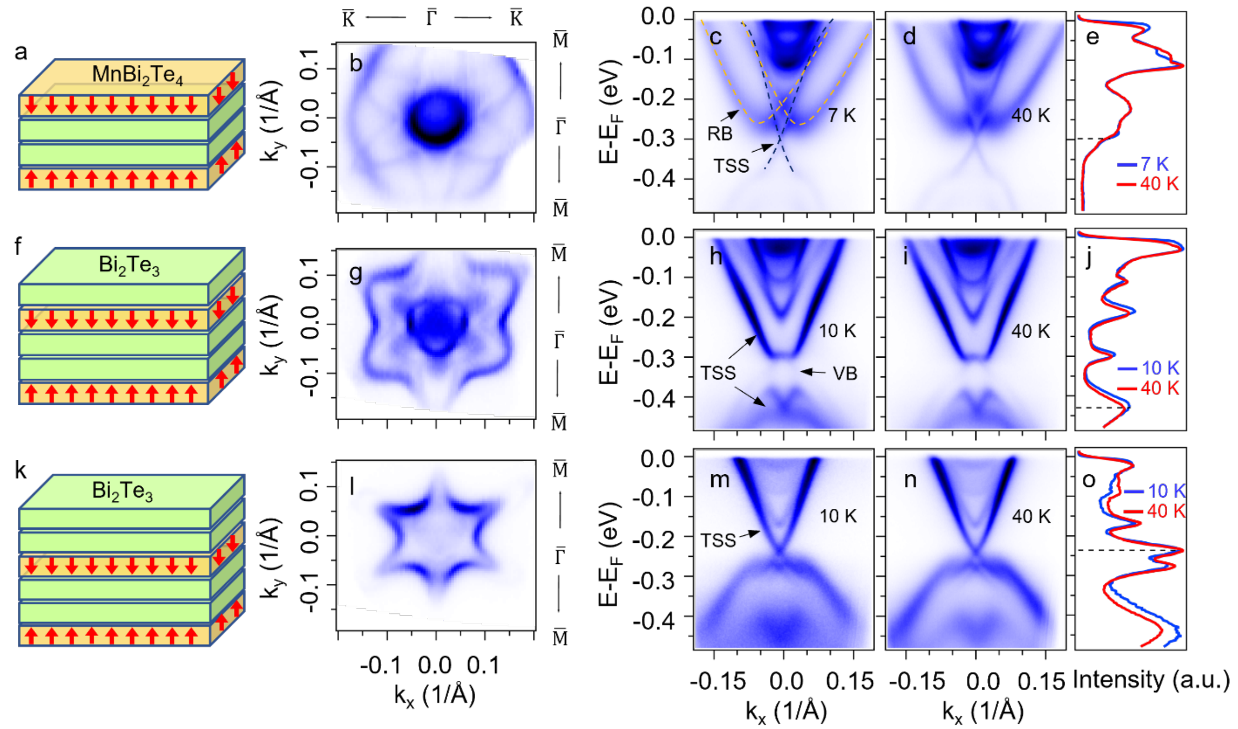

SI Figure 3. Electronic structure of antiferromagnetic $MnBi_6Te_{10}$. (a) Schematic drawing of the $MnBi_2Te_4$ (MBT) termination. (b) Fermi surface map of the MBT termination. (c, d) Energy-momentum spectra along the $\bar{\Gamma}-\bar{K}$ direction at (c) 7 K, and (d) 40 K. A pair of parabolic Rashba bands (RB) are observed on the MBT termination. Fitting the band dispersions to the standard Rashba model $E(k)^{\pm}=\hbar^2k^2/2m^{*}\pm\alpha_R|k|$ yields a substantial Rashba coupling constant α_R~3 eV.Å, which is comparable to some of the largest Rashba coupling constants such as α_R~3.85 eV.Å in BiTeI.[7] The hybridization between the RB and the TSS on the MBT termination is critical to the interpretation of various band structures.[8] Moreover, a slight band tilting is noticed in panel (c) and (d), which can be attributed to the stray field effect due to surface roughness. (e) Comparison of energy distribution curves at the $\bar{\Gamma}$ point. The black dashed line marks the Dirac point energy. The counterpart results for the 1-Bi_2Te_3 (1-BT) termination are plotted in (f-j). The counterpart results for the 2-BT termination are plotted in (k-o). One notable feature of the 1-BT ARPES data is that the original topological surface state is hybridized with a valence band, which

yields a hybridization gap. According to previous studies on $MnBi_4Te_7$,[9] this valence band originates mainly from the underlying MBT layer. This hybridization can only happen when MBT is the immediate underlying layer, which explains why this is not observed in the 2-BT ARPES data.

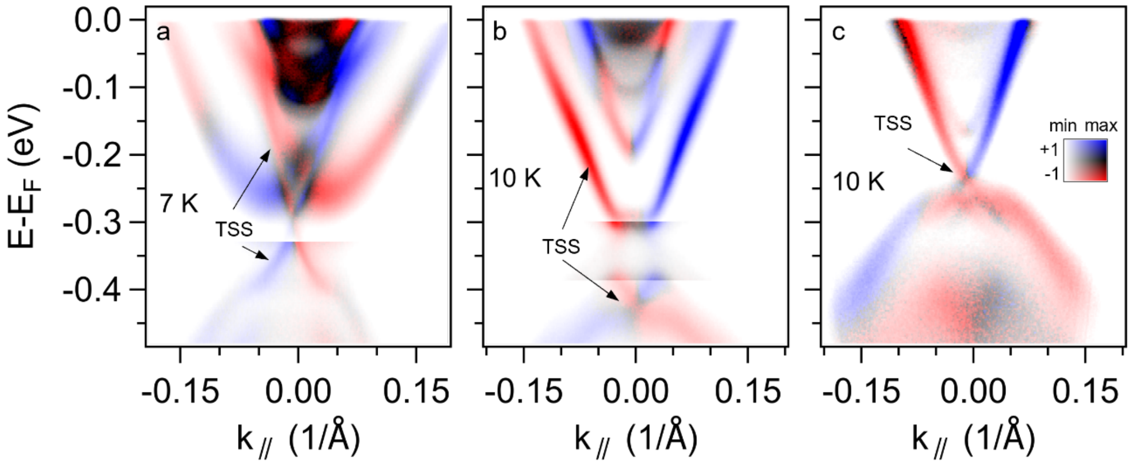

SI Figure 4. Circular dichroism in the ARPES spectra of antiferromagnetic $MnBi_6Te_{10}$. Circular dichroism maps are obtained for (a) $MnBi_2Te_4$ (MBT), (b) 1-Bi_2Te_3 (1-BT), and (c) 2-Bi_2Te_3 terminations, following the protocol detailed in SI Figure 1. The intensity scale is adjusted for the lower branch of the Dirac cone on the MBT termination, as well as for two spectral regions below -0.3 eV on the 1-BT termination.

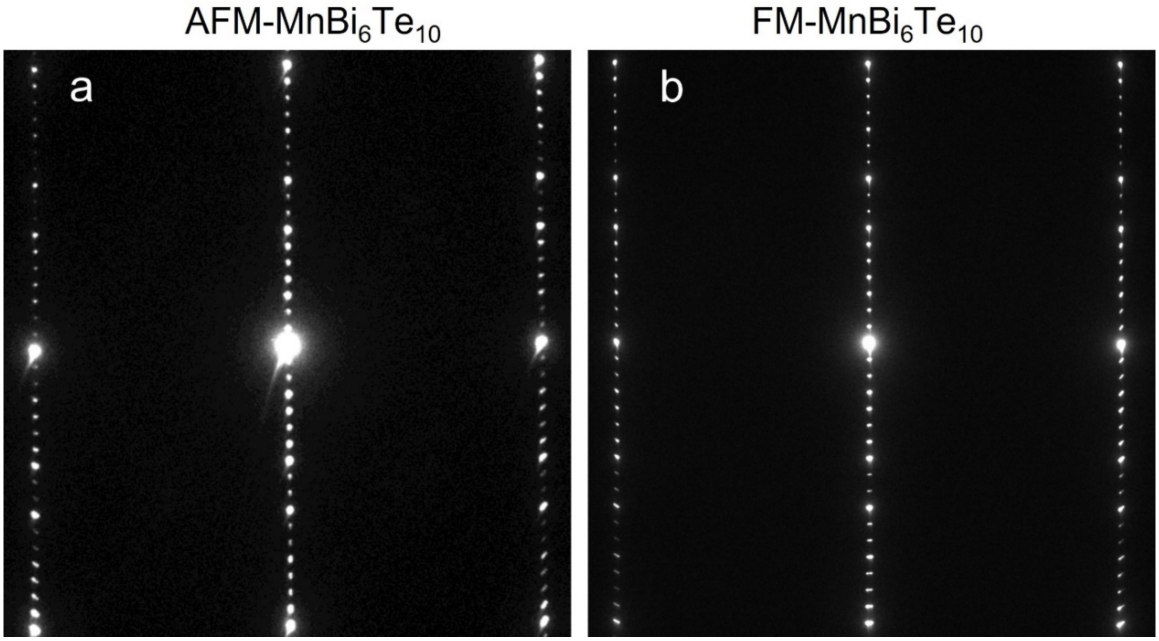

SI Figure 5. Selected area electron diffraction (SAED) patterns showing the single 1-6-10 phase for (a) AFM and (b) FM samples.

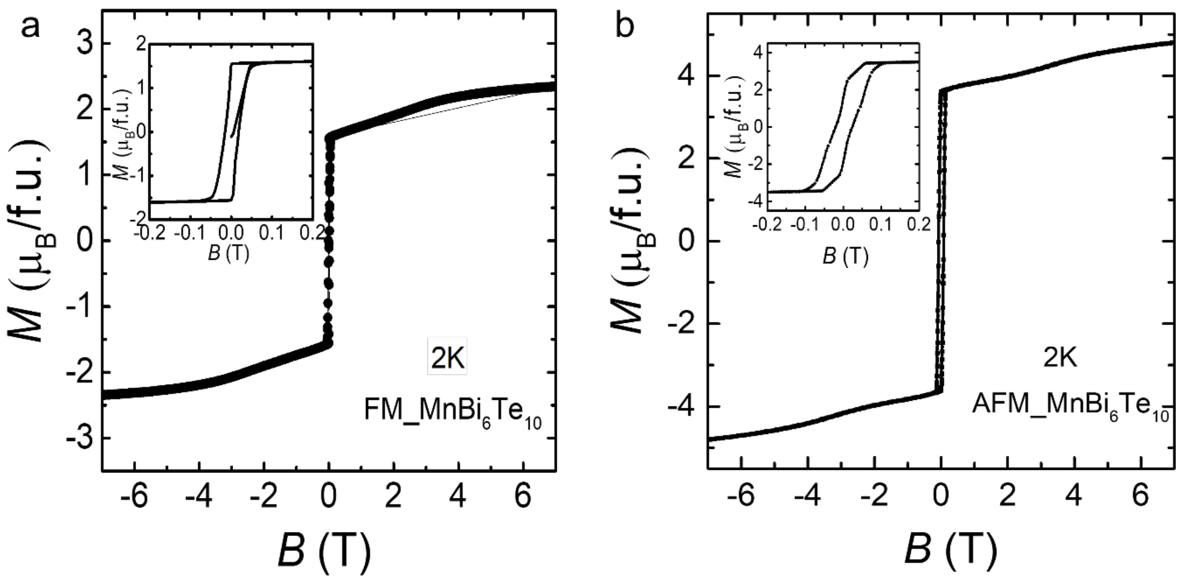

SI Figure 6. High-field magnetization measurements at 2 K. (**a**) Isothermal magnetization data measured up to 7 T with a magnetic field of 100 Oe applied along the *c*-axis for FM $MnBi_6Te_{10}$. (**b**) Same for AFM $MnBi_6Te_{10}$. The insets of (**a**) and (**b**) are zoomed-in views at low magnetic fields.

SI Table 1. Estimates of the chemical concentrations of $MnBi_6Te_{10}$ based on the scanning transmission electron microscopy – energy dispersive x-ray (STEM-EDX) analysis. Here norm. at. % stands for normalized atomic molar percentage. Note that the large error bar here is due to the poor signal-to-noise ratio in EDX measurements.

	**FM-$MnBi_6Te_{10}$**			**AFM-$MnBi_6Te_{10}$**		
**Element**	series	norm.at. %	Error in at. %	series	norm.at. %	Error in at. %
**Bi**	L-series	28.69	8.66	L-series	32.31	9.75
**Mn**	K-series	4.00	0.57	K-series	5.26	0.70
**Te**	K-series	67.31	6.37	K-series	62.43	6.13

SI Note 3: Magnetic interactions.

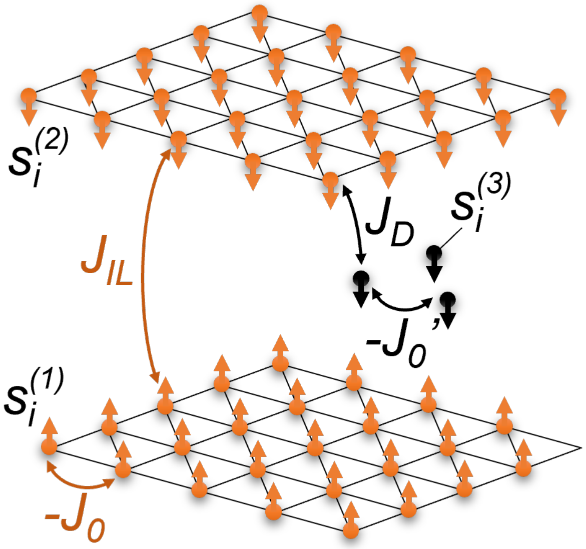

SI Figure 7. Magnetic interactions across two Mn sheets and one Mn migration layer. $\boldsymbol{S}_{\boldsymbol{i}}^{(\mathbf{1})}$, $\boldsymbol{S}_{\boldsymbol{i}}^{(\mathbf{2})}$, and $\boldsymbol{S}_{\boldsymbol{i}}^{(\mathbf{3})}$ represent the magnetic moments from the two Mn sheets (1, 2) and from the migration layer (3). We assume the *intralayer* coupling ($-J_0$, $-J_0'$) to be negative, favoring ferromagnetism. We assume the *interlayer* coupling (J_{IL}, J_D) to be positive, favoring antiferromagnetism.

We consider the interactions between the magnetic moments in the two original Mn sheets ($\boldsymbol{S}_{\boldsymbol{i}}^{(\mathbf{1})}$, $\boldsymbol{S}_{\boldsymbol{i}}^{(\mathbf{2})}$), and in a hypothesized migration layer $\boldsymbol{S}_{\boldsymbol{i}}^{(\mathbf{3})}$. Here the subscripts and superscripts stand for the site indices and sheet indices, respectively. The most general Hamiltonian can be written as follows.

$$H_{tot} = -\sum_{i.j}^{(1),(1)} J_{0,ij}\boldsymbol{S_i^{(1)}} \cdot \boldsymbol{S_j^{(1)}} - \sum_{i.j}^{(2),(2)} J_{0,ij}\boldsymbol{S_i^{(2)}} \cdot \boldsymbol{S_j^{(2)}}$$

$$+\sum_{i.j}^{(1),(2)} J_{IL,ij}\boldsymbol{S_i^{(1)}} \cdot \boldsymbol{S_j^{(2)}} - \sum_{i.j}^{(3),(3)} J'_{0,ij}\boldsymbol{S_i^{(3)}} \cdot \boldsymbol{S_j^{(3)}} \quad \text{(S1)}$$

$$+\sum_{i.j}^{(1),(3)} J_{D,ij}\boldsymbol{S_i^{(1)}} \cdot \boldsymbol{S_j^{(3)}} + \sum_{i.j}^{(2),(3)} J_{D,ij}\boldsymbol{S_i^{(2)}} \cdot \boldsymbol{S_j^{(3)}}$$

The *intralayer* interactions ($-J_{0,ij}$, $-J'_{0,ij}$) are generally assumed to be negative, which favors intralayer ferromagnetism. The *interlayer* interactions ($J_{IL,ij}, J_{D,ij}$) are assumed to be positive, which favors interlayer antiferromagnetism. These assumptions have been verified by experiments on $MnBi_{2n}Te_{3n+1}$ superlattices.[10,11] Considering the strong *c*-axis magnetic ordering, we simplify the magnetic moments to be Ising-like spins: $S_i^{(\alpha)} = \pm 1$. It is straightforward to evaluate the total energies for the ferromagnetic (FM) and antiferromagnetic (AFM) alignments between the two original Mn sheets.

- FM alignment: $S_i^{(1)} = S_i^{(2)} = 1, S_i^{(3)} = -1$. We obtain the following expression for the total energy.

$$E_{FM} = -\sum_{i.j}^{(1),(1)} J_{0,ij} - \sum_{i.j}^{(2),(2)} J_{0,ij} + \sum_{i.j}^{(1),(2)} J_{IL,ij} - \sum_{i.j}^{(3),(3)} J'_{0,ij}$$

$$-\sum_{i.j}^{(1),(3)} J_{D,ij} - \sum_{i.j}^{(2),(3)} J_{D,ij} \quad \text{(S2)}$$

Let $H_0 = \sum_{i,j}^{(1),(1)} J_{0,ij} = \sum_{i,j}^{(2),(2)} J_{0,ij}$ mark the intralayer coupling energy for the two original Mn sheets. Let $H'_0 = \sum_{i,j}^{(3),(3)} J'_{0,ij}$ mark the intralayer coupling energy for the migrated Mn ions. Furthermore, we use $H_{IL} = \sum_{i.j}^{(1),(2)} J_{IL,ij}$ and $H_D = \sum_{i.j}^{(1),(3)} J_{D,ij} = \sum_{i.j}^{(2),(3)} J_{D,ij}$ to represent the interlayer and the defect-induced coupling energies, respectively. Eqn. (S2) can be written as:

$$E_{FM} = -2H_0 + H_{IL} - H_0' - 2H_D \quad \text{(S3)}$$

- AFM alignment: $S_i^{(1)} = 1, S_i^{(2)} = S_i^{(3)} = -1$. We obtain the following expression.

$$E_{AFM} = -2H_0 - H_{IL} - H_0' \quad \text{(S4)}$$

Notably, the defect-induced coupling energies cancel each other due to the AFM alignment of the two Mn sheets. Combining Eqn. (S3) and (S4) yields Eqn. (1) in the main text.

$$E_{FM} - E_{AFM} = 2H_{IL} - 2H_D \quad \text{(S5)}$$

In this simplified three-layer Ising-spin picture, the magnetic ground state is determined by the competition between the interlayer coupling energy H_{IL} and the defect-induced coupling energy H_D. Most importantly, when $H_{IL} > H_D$ the AFM alignment of the two original Mn sheets is the favorable configuration; when $H_{IL} < H_D$ the FM alignment is favorable. Applying this model to the superlattice series $MnBi_{2n}Te_{3n+1}$, the interlayer coupling $J_{IL,ij}$ is expected to be progressively weaker for increasing superlattice order n, which is fully consistent with experimental observations.[10,11] A finite defect-induced ferromagnetic coupling $J_{D,ij}$, as well as other non-Ising-like interactions, can lead to a favorable FM configuration for higher-order superlattices ($n \geq 4$).[12]

Eqn. (S5) is also applicable when the defect-induced coupling J_D becomes FM. In this case, we can simply replace J_D with $-J_D$. In the FM alignment, $S_i^{(1)} = S_i^{(2)} = S_i^{(3)} = 1$; in the AFM alignment, $S_i^{(1)} = 1, S_i^{(2)} = S_i^{(3)} = -1$. Eqn. (S3)~(S5) will be unchanged. Therefore, our understanding of defect-induced delicate ferromagnetism is valid regardless of the sign of J_D. In fact, this will resolve the apparent discrepancy between our theoretical picture and the one in Ref.[13].

SI Note 4: Determining the Mn density ratio between different layers

Notably, H_{IL} involves two magnetic ions from the original Mn sheets, and is hence proportional to n_o^2, where n_o represents the density of the Mn ions in the original Mn sheets. H_D involves one Mn ion in the original Mn sheets and one migrated Mn ion, and is hence proportional to $n_o n_m$, where n_m represents the density of migrated Mn ions. If we assume that the migrated Mn ions are spread evenly among all Bi sites, the chemical formula will be $Mn_{1\text{-}y\text{-}6x}(Bi_{1\text{-}x}Mn_x)_6Te_{10}$. Here we adopt the convention in Ref.[14], where x stands for the proportion of migrated Mn ions and y stands for the proportion of Mn vacancies. By measuring the saturated magnetic moments at low field (M_1) and high field (M_2), one may derive the values of x and y.

$$M_1 = m_0(1 - y - 6x - 6x) \tag{S6}$$

$$M_2 = m_0(1 - y) \tag{S7}$$

Here m_0 is the saturated moment for one Mn ion (4.6~4.8 μ_B). It is straightforward to derive the ratio of n_m/n_o.

$$n_m/n_o = 6x/(1 - y - 6x) = (M_2 - M_1)/(M_2 + M_1) \tag{S8}$$

It is important to note that the expression of $(M_2 - M_1)/(M_2 + M_1)$ can be generalized to all $Mn_{1\text{-}y\text{-}2nx}(Bi_{1\text{-}x}Mn_x)_{2n}Te_{3n+1}$ materials, allowing us to directly evaluate the balance between migrated Mn ions and those in the original Mn sheets. In our work, we find that in FM $MnBi_6Te_{10}$ this ratio is close to 0.2 ($M_1 = 1.58$ $\mu_B/f.u.$, $M_2 = 2.36$ $\mu_B/f.u.$), yet in AFM $MnBi_6Te_{10}$ this ratio is 0.13 ($M_1 = 3.67$ $\mu_B/f.u.$, $M_2 = 4.8$ $\mu_B/f.u.$). Moreover, the value of M_2 measured at $7\,T$ is most likely an underestimate for FM $MnBi_6Te_{10}$. The realistic value of M_2 for FM

$MnBi_6Te_{10}$ can be even higher, leading to a higher n_m/n_o ratio. The value of M_2 for AFM $MnBi_6Te_{10}$ is already at the maximum (4.8 $\mu_B/f.u.$).

We would like to comment on the comparison between magnetization measurements and STEM-EDX measurements regarding the Mn vacancy density (y). STEM-EDX (SI Table 1) measurements yield that there are 24% fewer Mn atoms in FM $MnBi_6Te_{10}$, as compared to AFM $MnBi_6Te_{10}$. Assuming Mn vacancies are negligible in AFM $MnBi_6Te_{10}$, as the value of M_2 already reaches the maximum 4.8 $\mu_B/f.u.$ at high fields, the STEM-EDX results will lead to $y = 0.24$ for FM $MnBi_6Te_{10}$. In the meantime, using Eqn. (S7) we would obtain $y = 0.51$ for FM $MnBi_6Te_{10}$. While STEM-EDX analysis is usually subject to large uncertainties, this discrepancy is beyond what uncertainties can explain. We attribute this discrepancy to the fact that M_2 at 7 T for FM $MnBi_6Te_{10}$ is not quite saturated yet. The fully saturated M_2 for FM $MnBi_6Te_{10}$ will be higher, leading to a higher n_m/n_o ratio which still supports our conclusion in the main text, and a lower y which will reconcile with the STEM-EDX results.

Reference

(1) Ma, X.-M.; Chen, Z.; Schwier, E. F.; Zhang, Y.; Hao, Y.-J.; Kumar, S.; Lu, R.; Shao, J.; Jin, Y.; Zeng, M.; Liu, X.-R.; Hao, Z.; Zhang, K.; Mansuer, W.; Song, C.; Wang, Y.; Zhao, B.; Liu, C.; Deng, K.; Mei, J.; Shimada, K.; Zhao, Y.; Zhou, X.; Shen, B.; Huang, W.; Liu, C.; Xu, H.; Chen, C. Hybridization-Induced Gapped and Gapless States on the Surface of Magnetic Topological Insulators. *Phys. Rev. B* **2020**, *102* (24), 245136. https://doi.org/10.1103/PhysRevB.102.245136.

(2) Lu, R.; Sun, H.; Kumar, S.; Wang, Y.; Gu, M.; Zeng, M.; Hao, Y.-J.; Li, J.; Shao, J.; Ma, X.-M.; Hao, Z.; Zhang, K.; Mansuer, W.; Mei, J.; Zhao, Y.; Liu, C.; Deng, K.; Huang, W.; Shen, B.; Shimada, K.; Schwier, E. F.; Liu, C.; Liu, Q.; Chen, C. Half-Magnetic Topological Insulator with Magnetization-Induced Dirac Gap at a Selected Surface. *Phys. Rev. X* **2021**, *11* (1), 011039. https://doi.org/10.1103/PhysRevX.11.011039.

(3) Shikin, A. M.; Estyunin, D. A.; Klimovskikh, I. I.; Filnov, S. O.; Schwier, E. F.; Kumar, S.; Miyamoto, K.; Okuda, T.; Kimura, A.; Kuroda, K.; Yaji, K.; Shin, S.; Takeda, Y.; Saitoh, Y.; Aliev, Z. S.; Mamedov, N. T.; Amiraslanov, I. R.; Babanly, M. B.; Otrokov, M. M.; Eremeev, S. V.; Chulkov, E. V. Nature of the Dirac Gap Modulation and Surface Magnetic Interaction in Axion Antiferromagnetic Topological Insulator $MnBi_2Te_4$. *Sci Rep* **2020**, *10* (1), 13226. https://doi.org/10.1038/s41598-020-70089-9.

(4) Wang, G.; Zhu, X.-G.; Sun, Y.-Y.; Li, Y.-Y.; Zhang, T.; Wen, J.; Chen, X.; He, K.; Wang, L.-L.; Ma, X.-C.; Jia, J.-F.; Zhang, S. B.; Xue, Q.-K. Topological Insulator Thin Films of Bi_2Te_3 with Controlled Electronic Structure. *Advanced Materials* **2011**, *23* (26), 2929–2932. https://doi.org/10.1002/adma.201100678.

(5) Xiong, H.; Sobota, J. A.; Yang, S.-L.; Soifer, H.; Gauthier, A.; Lu, M.-H.; Lv, Y.-Y.; Yao, S.-H.; Lu, D.; Hashimoto, M.; Kirchmann, P. S.; Chen, Y.-F.; Shen, Z.-X. Three-Dimensional Nature of the Band Structure of $ZrTe_5$ Measured by High-Momentum-Resolution Photoemission Spectroscopy. *Phys. Rev. B* **2017**, *95* (19), 195119. https://doi.org/10.1103/PhysRevB.95.195119.

(6) Zhang, P.; Richard, P.; Qian, T.; Xu, Y.-M.; Dai, X.; Ding, H. A Precise Method for Visualizing Dispersive Features in Image Plots. *Review of Scientific Instruments* **2011**, *82* (4), 043712. https://doi.org/10.1063/1.3585113.

(7) Ishizaka, K.; Bahramy, M. S.; Murakawa, H.; Sakano, M.; Shimojima, T.; Sonobe, T.; Koizumi, K.; Shin, S.; Miyahara, H.; Kimura, A.; Miyamoto, K.; Okuda, T.; Namatame, H.; Taniguchi, M.; Arita, R.; Nagaosa, N.; Kobayashi, K.; Murakami, Y.; Kumai, R.; Kaneko, Y.; Onose, Y.; Tokura, Y. Giant Rashba-Type Spin Splitting in Bulk BiTeI. *Nature Mater* **2011**, *10* (7), 521–526. https://doi.org/10.1038/nmat3051.

(8) Yan, C.; Fernandez-Mulligan, S.; Mei, R.; Lee, S. H.; Protic, N.; Fukumori, R.; Yan, B.; Liu, C.; Mao, Z.; Yang, S. Origins of Electronic Bands in the Antiferromagnetic Topological Insulator $MnBi_2Te_4$. *Phys. Rev. B* **2021**, *104* (4), L041102. https://doi.org/10.1103/PhysRevB.104.L041102.

(9) Wu, X.; Li, J.; Ma, X.-M.; Zhang, Y.; Liu, Y.; Zhou, C.-S.; Shao, J.; Wang, Q.; Hao, Y.-J.; Feng, Y.; Schwier, E. F.; Kumar, S.; Sun, H.; Liu, P.; Shimada, K.; Miyamoto, K.; Okuda, T.; Wang, K.; Xie, M.; Chen, C.; Liu, Q.; Liu, C.; Zhao, Y. Distinct Topological Surface States on the Two Terminations of $MnBi_4Te_7$. *Phys. Rev. X* **2020**, *10* (3), 031013. https://doi.org/10.1103/PhysRevX.10.031013.

(10) Klimovskikh, I. I.; Otrokov, M. M.; Estyunin, D.; Eremeev, S. V.; Filnov, S. O.; Koroleva, A.; Shevchenko, E.; Voroshnin, V.; Rybkin, A. G.; Rusinov, I. P.; Blanco-Rey, M.; Hoffmann, M.; Aliev, Z. S.; Babanly, M. B.; Amiraslanov, I. R.; Abdullayev, N. A.; Zverev, V. N.; Kimura, A.; Tereshchenko, O. E.; Kokh, K. A.; Petaccia, L.; Di Santo, G.; Ernst, A.; Echenique, P. M.; Mamedov, N. T.; Shikin, A. M.; Chulkov, E. V. Tunable 3D/2D Magnetism in the $(MnBi_2Te_4)(Bi_2Te_3)_m$ Topological Insulators Family. *npj Quantum Mater.* **2020**, *5* (1), 54. https://doi.org/10.1038/s41535-020-00255-9.

(11) Hu, C.; Ding, L.; Gordon, K. N.; Ghosh, B.; Tien, H.-J.; Li, H.; Linn, A. G.; Lien, S.-W.; Huang, C.-Y.; Mackey, S.; Liu, J.; Reddy, P. V. S.; Singh, B.; Agarwal, A.; Bansil, A.; Song, M.; Li, D.; Xu, S.-Y.; Lin, H.; Cao, H.; Chang, T.-R.; Dessau, D.; Ni, N. Realization of an Intrinsic Ferromagnetic Topological State in $MnBi_8Te_{13}$. *Science Advances* **2020**, *6* (30), eaba4275. https://doi.org/10.1126/sciadv.aba4275.

(12) Tokura, Y.; Yasuda, K.; Tsukazaki, A. Magnetic Topological Insulators. *Nat Rev Phys* **2019**, *1* (2), 126–143. https://doi.org/10.1038/s42254-018-0011-5.

(13) Sitnicka, J.; Park, K.; Skupiński, P.; Grasza, K.; Reszka, A.; Sobczak, K.; Borysiuk, J.; Adamus, Z.; Tokarczyk, M.; Avdonin, A.; Fedorchenko, I.; Abaloszewa, I.; Turczyniak-Surdacka, S.; Olszowska, N.; Ko\lodziej, J.; Kowalski, B. J.; Deng, H.; Konczykowski, M.; Krusin-Elbaum, L.; Wo\loś, A. Systemic Consequences of Disorder in Magnetically Self-Organized Topological $MnBi_2Te_4/(Bi_2Te_3)_n$ Superlattices. *2D Mater.* **2021**, *9* (1), 015026. https://doi.org/10.1088/2053-1583/ac3cc6.

(14) Lai, Y.; Ke, L.; Yan, J.; McDonald, R. D.; McQueeney, R. J. Defect-Driven Ferrimagnetism and Hidden Magnetization in $MnBi_2Te_4$. *Phys. Rev. B* **2021**, *103* (18), 184429. https://doi.org/10.1103/PhysRevB.103.184429.
